# Methicillin-resistant *Staphylococcus aureus* not detected in Swedish nucleus and multiplying pig herds

**DOI:** 10.1080/20008686.2017.1313068

**Published:** 2017-04-12

**Authors:** Helle Ericsson Unnerstad, Helene Wahlström, Benedicta Molander, Björn Bengtsson

**Affiliations:** ^a^ Department of Animal Health and Antimicrobial Strategies, National Veterinary Institute, Uppsala, Sweden; ^b^ Department of Disease Control and Epidemiology, National Veterinary Institute, Uppsala, Sweden; ^c^ Farm and Animal Health, Staffanstorp, Sweden

**Keywords:** MRSA, *Staphylococcus aureus*, CC398, pig

## Abstract

**Introduction:** Livestock-associated methicillin-resistant Staphylococcus aureus (LA-MRSA) has emerged among pigs in many countries. MRSA in the pig population constitute a reservoir with risk for transmission to humans in close contact with pigs. Absence of MRSA in the top of the breeding pyramid would prevent spread to the rest of the pig population. The aim of this study was to investigate the occurrence of MRSA in nucleus and multiplying pig herds in Sweden.

**Materials and methods:** All nucleus and multiplying pig herds in Sweden present in 2011 (*n *= 53) and 2014 (*n *= 39) were sampled for MRSA.

**Results and discussion:** MRSA was not detected either in 2011 or in 2014. That MRSA was not detected in the top of the breeding pyramid indicates a favourable MRSA situation in the Swedish pig population.

**A** **bbreviations:** MRSA: methicillin-resistant *Staphylococcus aureus*; LA-MRSA: livestock-associated MRSA; CC: clonal complex

## Introduction

Methicillin-resistant *Staphylococcus aureus* (MRSA) is resistant to all beta-lactam antibiotics and infections cannot be treated with antibiotics usually used for staphylococcal infections. MRSA has long been a problem in human healthcare but also through transmission in the community.[1] During the last decade, livestock-associated MRSA (LA-MRSA) in Europe, mainly MRSA of clonal complex (CC) 398, has emerged among pigs in many countries.[2] In Denmark, the prevalence of LA-MRSA at herd level was 63% in breeding herds and 68% in slaughter herds in 2014.[3] In a Norwegian study in 2015, 0.5% of sampled nucleus, multiplier and finishing herds were positive for LA-MRSA.[4] Norway has a national strategy including a ‘search-and-destroy’ policy with outbreak investigations, contact tracing, surveillance and stamping out of positive herds.[5] Typically, pigs carry MRSA without symptoms, although there are rare reports of clinical disease.[6] Thus, the main problem with MRSA in pigs is the risk of spread to humans. People in close contact with pigs, i.e. pig farmers, veterinarians, pig transporters and slaughterhouse workers, are at risk of being colonised.[7] In countries with low prevalence of MRSA in humans, a reservoir in pigs may give a significant contribution to the human MRSA burden.[8]

Cross breeding is used in pig production with the aim to increase litter sizes and growth performance. Thus, the production is shaped like a pyramid, where a few pure bred nucleus herds produce pure bred animals that are sold to a few multiplying herds that produce cross bred sows for production of growers in a large number of piglet producing herds. Trade of live animals is considered a risk factor for MRSA in pig populations.[9] Thus, absence of MRSA in the top of the breeding pyramid would be a first prerequisite for preventing spread of MRSA by trade of live animals to the rest of the pig population. The genetic base for pig production in Sweden is concentrated to nucleus and multiplying herds and these herds provide the production herds with breeding stock. Trade of breeding animals between production herds is, however, uncommon in Sweden and import of pigs to Sweden is very limited. Moreover, imported breeding pigs are quarantined and tested for MRSA before being introduced to Swedish herds. Imported semen is also tested for MRSA before use in Swedish herds. Introduction of MRSA to nucleus and multiplying herds would lead to a substantial risk of spread further down in the breeding pyramid. It is therefore of importance to have knowledge of the MRSA situation in herds in the top of the breeding pyramid. The aim of this study was to investigate the occurrence of MRSA in nucleus and multiplying pig herds in Sweden.

## Materials and methods

### Herds included

All nucleus and multiplying herds present were sampled in 2011 (*n* = 53) and in 2014 (*n* = 39), meaning that the entire top of the breeding pyramid was included. The sampling was conducted anonymously, meaning that the results could not be connected to a certain herd. The sampled herds were of different sizes, ranging from 20 to 100 pens of weaned pigs per herd and 10 to 100 pigs per pen.

### Sampled pigs

Based on studies indicating that the highest MRSA prevalence is expected to be found in growing pigs shortly after weaning,[10–12] weaned pigs in the age group 5–12 weeks were chosen for sampling. Due to practical and economic reasons, it was decided to sample six pigs per pen and analyse these samples as one pooled sample. The intention was to sample pigs from 15 pens in each herd. In this way 90 weaned pigs per herd would be sampled. Pigs older than the intended 12 weeks were sampled in one herd in 2011 and in five herds in 2014, since no pigs 5–12 weeks old were present. In 2011, eight, 14 and 17 pens, respectively, were sampled in three herds. In 2014 only four pens were sampled in one herd. Altogether, 4734 pigs in 789 pens were sampled in 2011, and 3444 pigs in 574 pens in 2014.

### Assumptions

An average herd was assumed to have 50 pens with 25 weaned pigs in the desired age group in each pen based on information from Farm and Animal Health. It was assumed that, in a positive herd, 30% of the pens would be positive (contain colonised pigs) and that 50% of the pigs in a positive pen would be colonised. The sampling sensitivity, i.e. the probability that MRSA would be captured in the swab sample given that the tested pig is colonised, was assumed to be 50%, the analytical sensitivity to be 50% and risk of decreased sensitivity due to pooling was not considered. Since exact information was not available, these estimates were based on expert opinion and they were considered to be conservative.

### Sensitivity calculations

The sensitivity for the sampling strategy was calculated as follows:

The sensitivity on the individual animal level (*SeInfTest*) was calculated as:

(1) 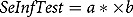

where *a *= the sampling sensitivity and *b *= the analytical sensitivity.

The sensitivity on the box level (*SeBox)*, was calculated as 1 − *A*, where *A* is the probability that there are no test positive pigs in the box, given the box is positive.[13]

(2) 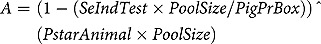

where *PoolSize* = number of samples in the pooled sample, *PigPrBox* = number of pigs in the box and *PstarAnimal* is the prevalence of MRSA colonised pigs in the box. The sensitivity on the herd level, i.e. the probability of getting at least one positive sample if the herd is positive, was calculated in a similar way.

Given the assumptions and calculations above, and if 15 pens per herd are sampled, there was a 93% probability of detecting MRSA if the herd was positive. This was considered sufficient.

### Sample collection and laboratory analyses

Sampling was done by rubbing the skin behind one ear with a sterile compress. One compress was used to sample six pigs in each pen. During sampling, mouth mask and sterile gloves were worn and the gloves were changed between each pen. After sampling, each compress was put in a sterile plastic jar and sent to the laboratory by mail.

The samples were pre-enriched in Müller-Hinton broth with 6.5% NaCl in 37°C for 16–20 h. One ml of the pre-enrichment was mixed with 9 ml of selective enrichment of tryptic soy broth with 3.5 mg l^–1^ cefoxitin and 75 mg l^–1^ aztreonam and incubated in 37°C for 16–20 h. Ten µl of the selective enrichment broth were plated on selective agar (Brilliance MRSA, Oxoid, Wesel, Germany) and 10 µl were plated on bovine blood agar. Suspected colonies were further investigated by PCR for detection of the *mec*A or *mecC* genes.

## Results and discussion

All samples in the two present studies were negative for MRSA. Using the present study design the herd sensitivity is 93%. This means that there is a 7% probability that, given that only one positive herd is present, MRSA will not be detected. However, this probability decreases if more herds are positive. If two or three herds are positive, the probability of not detecting at least one of them will decrease to 0.07 (0.5%) and 0.07 (0.03%). Furthermore, since the design prevalences used (between and within pen prevalences) and the sampling sensitivity was not known, estimates were used. These estimates are considered to be conservative to ensure that the sensitivity of the surveillance was not overestimated. However, not taking the effect of pooling into account will slightly overestimate the sensitivity. As it was considered unlikely that only one herd would be positive, the overall conclusion was that if MRSA was present in the top of the breeding pyramid, it would most probably have been detected in these surveys.

MRSA has only been found in pigs in Sweden once [14] and the preventive measures taken by the industry have probably contributed to the presumed low prevalence. In the present study, all Swedish nucleus and multiplying pig herds were screened for MRSA in 2011 and 2014, without findings of MRSA. The negative outcome confirms the results of earlier screening studies in which MRSA was not found in nasal swabs from fattening pigs in 100 herds in 2006–2007, or in dust samples from 202 production and breeding herds in 2008.[15,16] In 2010, MRSA was detected in one pool of five nasal swabs from one of 191 sampled fattening herds in an anonymous study.[17] The absence of MRSA at the top of the breeding pyramid indicates that Sweden has a favourable situation concerning MRSA in the pig population. However, the situation can easily change and stringency in biosecurity and control of trade with live animals are of utmost importance.

In conclusion, Swedish nucleus and multiplying pig herds were found to be negative for MRSA in both 2011 and 2014.

## References

[CIT0001] Grundmann H, Aires-de-Sousa M, Boyce J (2006). Emergence and resurgence of meticillin-resistant Staphylococcus aureus as a public-health threat. Lancet.

[CIT0002] Verkade E, Kluytmans J. (2014). Livestock-associated Staphylococcus aureus CC398: animal reservoirs and human infections. Infect Genet Evol.

[CIT0003] (2015). DANMAP 2014 – use of antimicrobial agents and occurrence of antimicrobial resistance in bacteria from food animals, food and humans in Denmark.

[CIT0004] NORM/NORM-VET 2015 (2016). Usage of antimicrobial agents and occurrence of antimicrobial resistance in Norway.

[CIT0005] Grøntvedt CA, Elstrøm P, Stegger M (2016). Methicillin-resistant Staphylococcus aureus CC398 in humans and pigs in Norway: a “one health” perspective on introduction and transmission. Clin Infect Dis.

[CIT0006] van der Wolf PJ, Rothkamp A, Junker K (2012). Staphylococcus aureus (MSSA) and MRSA (CC398) isolated from post-mortem samples from pigs. Vet Microbiol.

[CIT0007] Liu W, Liu Z, Yao Z (2015). The prevalence and influencing factors of methicillin-resistant Staphylococcus aureus carriage in people in contact with livestock: a systematic review. Am J Infect Control.

[CIT0008] (2009). EFSA: joint scientific report of ECDC, EFSA and EMEA on meticillin resistant Staphylococcus aureus (MRSA) in livestock, companion animals and foods. EFSA-Q-2009-00612 (EFSA Scientific Report (2009) 301, 1–10) and EMEA/CVMP/SAGAM/62464/2009.

[CIT0009] EFSA: European Food Safety Authority (2010). Analysis of the baseline survey on the prevalence of methicillin-resistant Staphylococcus aureus (MRSA) in holdings with breeding pigs, in the EU, 2008, part B: factors associated with MRSA contamination of holdings; on request from the European Commission. EFSA J.

[CIT0010] Dewaele I, Messens W, De Man I (2011). Sampling, prevalence and characterization of meticillin-resistant Staphylococcus aureus on two Belgian pig farms. Vet Sci Dev.

[CIT0011] Weese JS, Zwambag A, Rosendal T (2011). Longitudinal investigation of methicillin-resistant Staphylococcus aureus in piglets. Zoonoses Public Health.

[CIT0012] Smith TC, Male MJ, Harper AL (2009). Methicillin-resistant Staphylococcus aureus (MRSA) strain ST398 is present in midwestern U.S. swine and swine workers. Plos One.

[CIT0013] MacDiarmid SC. (1988). Future options for brucellosis surveillance in New Zealand beef herds. N Z Vet J.

[CIT0014] Swedres-Svarm 2014 (2015). Consumption of antibiotics and occurrence of antibiotic resistance in Sweden.

[CIT0015] Eliasson-Selling L, Grönlund Andersson U, Greko C (2008). Screening for methicillin-resistant Staphylococcus aureus (MRSA) in Swedish pigs.

[CIT0016] EFSA: European Food Safety Authority (2009). Analysis of the baseline survey on the prevalence of methicillin-resistant Staphylococcus aureus (MRSA) in holdings with breeding pigs, in the EU, 2008, part A: MRSA prevalence estimates; on request from the European Commission. EFSA J.

[CIT0017] SVARM 2010 (2011). Swedish Veterinary Antimicrobial Resistance Monitoring.

